# It’s All Up to My Fellow Citizens. Descriptive Norms as a Decisive Mediator in the Relationship Between Infrastructure and Mobility Behavior

**DOI:** 10.3389/fpsyg.2020.610343

**Published:** 2021-02-10

**Authors:** Philipp Rollin, Sebastian Bamberg

**Affiliations:** Faculty of Social Sciences, University of Applied Sciences, Bielefeld, Germany

**Keywords:** mobility behavior, descriptive norms and behaviors, behavior change, pop-up bike lane, social norms, street design

## Abstract

Following the implementation of temporary pop-up bike lanes in Berlin, traffic counts by the city administration show an increased number of cyclists. This present paper aims to understand reasons behind this observation. To this end, we focus on the role of mobility-related descriptive social norms as mediators of this effect. Results from one correlational and two experimental online studies are reported. The correlational study confirms the expected association of mobility-related descriptive social norms and self-reported mobility behavior. Moreover, it demonstrates that, as expected, mobility-related descriptive social norms reliably reflect differences in cities’ objective transport structure and mediate the impact of these infrastructural differences on mobility behavior. Results from two online experiments provide additional causal evidence that participants use the visual cues provided by manipulated photos to form their perceived mobility-related descriptive social norms. Furthermore, the second online experiment provides evidence that the combination of infrastructural cues and observable mobility behavior has the strongest impact on perceived mobility-related descriptive social norms.

## Introduction

**FIGURE 1:**
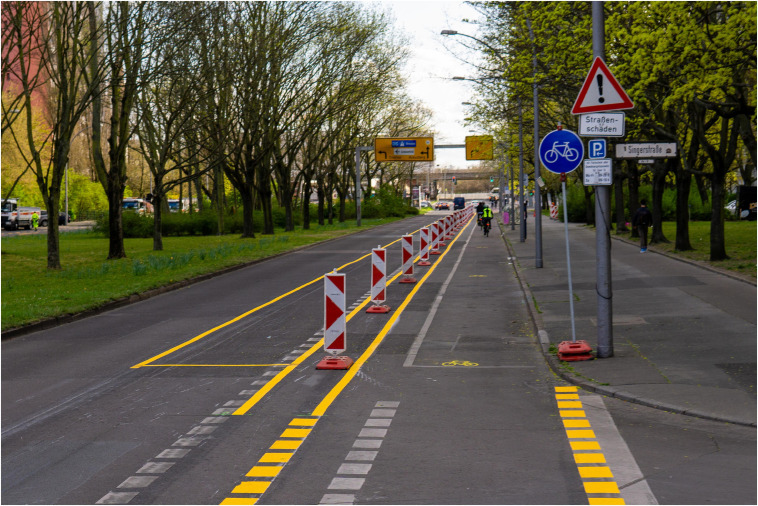
A “pop-up bike lane” in Berlin-Kreuzberg, built at thebeginning of the corona crisis in 2020 (source: Peter Broytman, qimby.net).

At the beginning of the corona crisis, German cities such as Düsseldorf, München, or Berlin (Deutschlandfunk, 2020) surprised the public by introducing temporary bicycle lanes. The aim of these lanes was to enable cyclists to keep the recommended minimum distance of 1.5 m, thereby enabling more people to cycle in order to avoid using public transportation as a means of reducing the infection risk (Infravelo, 2020). As can be seen in Figure 1, these so-called “pop-up bike lanes” were constructed with minimal investment costs: simple markers, paint, and a sign were used to transform a part of the former car lane into a new pop-up bike lane.

From a behavioral scientific point of view, the results of traffic counts conducted in Berlin after introducing pop-up bike lanes are impressive: the counting points show that in June 2020, 26.5% more bicycles were counted in the city than in the same period a year earlier (Tagesspiegel, 2020a). Obviously, the cheap investment in visually separating a part of the road from car traffic motivates more city residents to use the now “free” street space for cycling. This raises the question of which psychological processes mediate the impact of this simple and economic policy measure on individual transport means choice. Urban planners will likely argue that residents automatically perceive the separated street space as a new separated cycle path (“pop-up bike lane”), and that respective research indicates that the existence of separate cycle paths increases the perceived safety of cycling. Perceived safety in turn increases the intention of cycling as well as actual cycling (Handy et al., 2002; Pucher and Buehler, 2008; Goetzke and Rave, 2011; Frumkin et al., 2013). However, the reported direct empirical correlation of objective infrastructural conditions and cycling is low and says nothing about causality or the assumed underlying mechanisms of this causality (Ferdinand et al., 2012; Ding et al., 2018). Furthermore, another pop-up bike lane example in Berlin’s Kantstrasse also indicates that the relationship between objective infrastructural conditions and cycling may not be as direct and simple as assumed by urban planners. In this case, the residents do not use the new pop-up lane for cycling, but as additional free parking spots for their cars (Tagesspiegel, 2020b). Why then do people perceive and use the separated free road space for cycling in one case and as parking space in another case? From a behavioral scientific perspective, this observation raises the question of which situational framework and psychological processes mediate the relationship between objective infrastructure and behavior. In the present paper, we will answer this question by referring to the social norm concept or, more precisely, the concept of descriptive social norm as a potential mediator of the relationship between objective infrastructure and behavior. For this purpose, we will define the social norm concept and summarize research on the descriptive social norm—individual mobility behavior relationship as well as causal mechanisms assumed to underlie this relationship in the following section. Based on research hypotheses derived from the theoretical summary in the following sections, we will present the results of one correlational and two experimental studies empirically analyzing the mediating role of descriptive social norms. The last section discusses the theory- and practice-related implications of the presented results.

## The Social Norm Concept

The concept of social norm marks one cornerstone of theory building in psychology and sociology: The classical studies by Sherif (1936) and Asch (1956) have documented that people assimilate their perceptions and thoughts to social group norms because peers’ responses are a key source of information and social support. Influential theories like the theory of planned behavior (Ajzen, 1991) also underline the significance of perceived social norms as predictors of a wide range of intentions or behaviors. Furthermore, social identity theory (Tajfel, 1978) stresses the importance of social norms for understanding group-based behavior: Social norms define the behaviors expected from a prototypical “good” group member. Thus, activating the salience of personally essential group memberships increases the impact of social norms on individual behavior. A further important theoretical framework for the present paper is the differentiation of the general social norm concept introduced by Cialdini et al., 1990, 1991) and Kallgren et al. (2000) into the concepts of descriptive social norms (what most people do in a situation) and injunctive social norms (what behavior most people approve in a situation). Kashima et al. (2013) focus on acquiring social norms as social category learning. This means that people learn social information about community social norms from others with whom they are associated through social network ties.

### Descriptive Social Norm and Individual Mobility Behavior

Over the last years, a growing number of studies have started to analyze the impact of social norms on individual mobility behavior (e.g., Abou-Zeid et al., 2013; Kim et al., 2017). These studies focus on the association between a person’s perception of how frequently other individuals use a specific means of transport (car, cycling, public transport, or walking) for a specific purpose (e.g., shopping, commuting) and their own use of these means of transport for these purposes. For example, some studies found that people are more likely to cycle themselves if they perceive their peers as cyclists (Goetzke and Rave, 2011; Handy et al., 2014; Sherwin et al., 2014). However, how generalizable is this association between perceived social norm and individual behavior? A meta-analysis published by Gardner and Abraham (2007) reports a pooled medium-sized correlation between descriptive car use social norm and the individual car use (*r*_+_ = 0.36, *p* < 0.001). A similar result (*r*_+_ = 0.26, *p* < 0.05) is reported in the meta-analysis published by Lanzini and Khan (2017) and reported a pooled correlation of *r* + = 0.21 (*p* < 0.01) between a perceived non-car use descriptive social norm and individual cycling, public transport use, and walking. However, Hoffmann et al. (2017) report a small, statistically not significant negative correlation between the perceived descriptive car use social norm and individual car use (*r* + = −0.07) in their meta-analysis. Obviously, the correlational evidence of a reliable descriptive social norm and the individual mobility behavior relationship is ambiguous, which could be interpreted as indicating mediating causal mechanisms.

### Causal Mechanisms Mediating the Descriptive Social Norm–Behavior Relation

Cialdini et al. (1990, 1991) were the first to systematically analyze psychological processes mediating the perceived social norm–individual behavior relationship. They demonstrate that the situational activation of a respective social norm in people’s minds is a central precondition for its influence on individual behavior (Reno et al., 1993). For example, an antilitter norm will be more on the forefront of people’s minds when they see someone picking litter (which shows disapproval of littering, Cialdini et al., 1991) or simply see a norm stated on a sign (Winter et al., 2000; Cialdini, 2003). Furthermore, Cialdini et al. (1990, 1991) assume that descriptive social norms affect behavior because they provide information about which behavior is most common in a given situation. Thus, the behavioral impact of descriptive social norms is based on people’s tendency to reason that if, in the present situation, many people are doing this, it is probably a wise thing to do (Cialdini, 2007). Cialdini et al. (1990, 1991) also analyzed what happens when injunctive and descriptive norms are in conflict, for example, in a setting where it seems to be common practice to litter, even though littering is commonly disapproved of (discrepancy of descriptive and injunctive social norms). They found that such a conflict reduces the impact of injunctive norms and increases the probability that people follow the perceived descriptive norm: So when perceived norm-breaking behavior is visible, people are more likely to adapt to the perceived descriptive norm than to the supposedly injunctive norm. This line of research was expanded upon by Keizer et al. (2008; 2011). In the context of prohibitive littering, graffiti spraying, and bicycle parking, they found that visible traces of injunctive norm-violating behavior by others likewise inhibit the influence of injunctive norms represented, for example, by a sign explicitly prohibiting these behaviors. Their experimental results indicate that this inhibiting effect of observable norm-violating behavior traces is based on the fact that making a norm salient using a prohibition sign directs people’s attention not only toward this injunctive norm but also to the corresponding norm-violating descriptive norm, thereby enhancing the influence of these discrepant descriptive norms (so-called cross norm reversal effect). This assumption, i.e., that social norms are embedded in observable behavioral traces, has been demonstrated in the domain of eating behavior by Raghoebar et al. (2019). They use various photos of buffets and other similar instances of Dutch outside-the-home food contexts. It was tested whether these photos of physical cues stimulate certain eating norms, communicating what is socially accepted as usual and/or appropriate to eat in the examined contexts. The results of this photo study show that photos of a wide range of physical cues in food environments have the potential to communicate such eating-related descriptive and injunctive social norms. For instance, empty plates at a buffet convey the descriptive norm that many other guests have taken food and that it is, therefore, “normal” in this situation to take food from the plate. Inspired by this study, we present two online studies in this article using photomontages of streets with or without pop-up bike lanes to test whether such a temporary infrastructural measure is equally sufficient to make people perceive a specific descriptive, mobility-related norm and can therefore provide an explanation about the underlying psychological processes, mediating the impact of such a simple infrastructural measure on the mobility behavior of the residents.

## The Present Research

**FIGURE 2:**
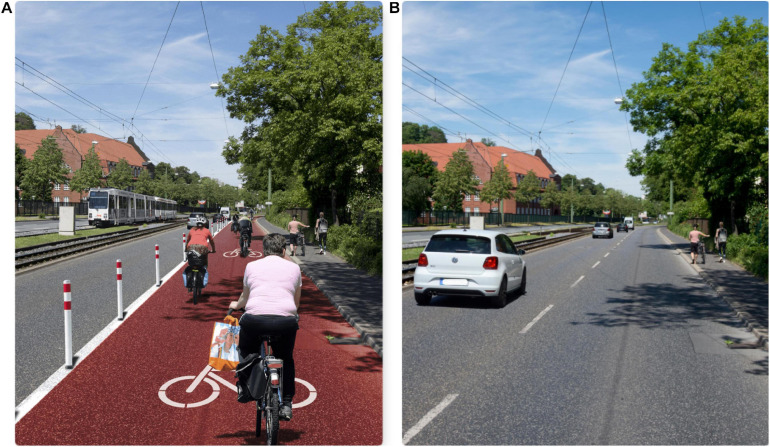
Photomontages used for the experimental online study 2. On the left, the descriptive norm “sustainability-oriented mobility behavior” **(A)**; on the right, the descriptive norm “car-oriented mobility behavior” **(B)**. (Photos: Michael Motyka).

In the first section, we report the results of a first online study providing a correlational test of our central hypothesis (H1). That means that people use perceived infrastructural conditions and the observable travel behavior of their fellow citizens resulting from these infrastructural conditions as sources for the construction of their mobility-related descriptive social norms (based on their perception of what transportation means most fellow citizens view as the adequate “usual” choice for everyday trips). For this purpose, we assessed participants’ perceived mobility-related descriptive social norms as well as their self-reported personal transportation means use in six different city types (e.g., cycling-oriented city vs. car-oriented city). We expect that the cities’ different objective transport infrastructures are reflected in respective differences in participants’ perceived mobility-related descriptive social norms, as well as their self-reported transportation means. In the next two sections, we use the context of pop-up bike lanes for conducting two online experiments for getting a better understanding of the psychological processes underlying the assumed effect of infrastructural features on residents’ cycling behavior. More precisely, we assume that the eye-catching features of the pop-up bike lanes (markers and paint, see Figure 1), together with the observation that other residents actively use the marked free street space for cycling, lead to the perception that, on this street, cycling is now a normal/adequate mobility behavior (perceived mobility-related descriptive social norm). In the first online experiment, we will present participants with two pictures of the same street used by cars, cyclists, and a streetcar. However, one picture includes a pop-up bike lane and the other no pop-up bike lane (Figure 2) to test this assumption empirically. In line with the theoretical argument that social norms are imbedded in environmental cues, we assume that adding a pop-up bike lane as a new infrastructural feature to the picture will cause a significantly stronger perceived descriptive cycling social norm (H2). The experimental context also allows to test an alternative theoretical explanation of differences in the perceived descriptive social norms: From Bem’s (1972) self-perception theory, one can derive the hypothesis that instead of a different objective infrastructural condition, different perceived descriptive social norms mainly reflect participants’ own past transport means use (H3). Inspired by the Raghoebar et al. (2019), we analyze whether changes in the objective infrastructural condition (pop-up bike lane vs. no pop-up bike lane) or in the observable behavior (people showing mobility behavior vs. no people) are more important information sources for participants’ mobility-related descriptive social norm construction (H4) in the second online experiment. In the last section, we discuss the three studies’ insight, their possible practical significance, and future research recommendations.

### Study 1

As stated above, our first study primarily aims to test H1, namely, that differences in the objective transport infrastructures of cities are reflected in respective differences in participants’ perceived mobility-related descriptive social norms as well as their self-reported transportation means use. Furthermore, study 1 also aims to test the assumption that the impact of the objective transport infrastructure differences on individual mobility behavior is not direct, but only indirectly mediated through perceived mobility-related descriptive social norms.

In study 1, we use a typology developed by Klinger et al. (2013) to select the cities where, in a second step, we sampled the study participants. By using factor and cluster analyses of a set of 23 mainly objective infrastructural indicators obtained from 44 German cities, Klinger et al. (2013) were able to identify six city types characterized by clearly different infrastructural conditions (e.g., “cycling cities” and “car-oriented cities”). For our study, we randomly select two examples from the list of cities assigned to each city type. In a next step, we commissioned a commercial online access panel provider to conduct an online study with participants living in these 12 cities.

#### Procedure

At the beginning of the questionnaire, participants were informed that they were participating in a study aiming to assess citizens’ actual mobility behavior and their own, as well as their fellow citizens’ mobility needs. The instruction given before participants completed the items assessing their descriptive social norms was as follows: “Please note that when answering the following questions, you should think of your neighbors and your perception, which means modes of transport they normally use. These questions are not about your own mobility behavior.”

#### Measurement

We assessed participants’ perceived mobility-related descriptive social norm by asking them how frequently people living in their city district use the transportation means car, bicycle, public transport, and walking (“The people living in my city district use the car for commuting”) in three different scenarios (everyday, work, and shopping). Participants answered on a 6-point scale, whose answer categories are in line with those of the “Mobilität in Deutschland (MiD)–Mobility in Germany” survey from the federal ministry for transport and digital infrastructure (BMVI), which has been conducted at irregular intervals since 2002 and offers the following response options: almost never, less than once a month, less than three times a month, less three times a week, over three times a week, and almost daily (BMVI, 2018). The same item format and response scale were used for assessing participants self-reported transport means use for the three scenarios commuting, shopping, and other everyday trips (“I use the car for commuting …”). Additionally, we assessed car availability and ownership of a seasonal public transport (PT) ticket as personal characteristics assumed to influence transport means use.

#### Sample

We commissioned the commercial access panel provider to acquire city subsamples of *n* = 60 participants in each of the following German cities: (1) Münster and Bremen (type cycling city), (2) Duisburg and Wuppertal (type car-oriented city), (3) Hamburg and Munich (type transit metropolises), (4) Bochum and Nürnberg (type transit cities with multimodal potential), (5) Frankfurt/M. and Stuttgart (type walking cities with multimodal potential), and (6) Dresden and Leipzig (type transit cities). For the definition and operationalization of the city types, we referred to the study by Klinger et al. (2013). The total sample consists of 725 participants. The median age is 45 years (range 18–80 years), 53.5% are females, 52.6% working full-time, 14.3% working part-time, 9.2% in education, 8.8% are jobless, and 15% are on pension.

#### Results

As the first analysis step, we built dummy variables representing participant’s membership to one of the six city types proposed by Klinger et al. (2013). We use these in the analyses as independent variables that represent infrastructural framework conditions, even though the interpretation of city types as indicators of infrastructural framework conditions is somewhat complicated, since they are based primarily, but not exclusively, on objective factors. In a second step, we constructed four index variables: eco-mobility-related descriptive social norm, car use-related descriptive social norm, individual car use, and individual eco-mobility. Cronbach’s α of all four index variables is >0.78. The means of the index variables assessing the perceived descriptive social norms and participants’ self-reported own mobility behavior confirm the expectation that Klinger’s six city types represent cities with very different infrastructural frame conditions: For example, participants living in the two car-oriented cities report the highest means for car norms and usage (Table 1).

**TABLE 1:** (Study 1) Labels, Cronbach’s α, means, SDs, and correlations of the descriptive norms and individual mobility behavior mean value indices (*N* = 725).

Complete Sample (*N* = 725)	M	SD	1	2	3
(1) Car use-related descriptive social norm (α = 0.79)	4.34	0.74			
(2) Eco-mobility-related descriptive social norm (α = 0.85)	3.78	0.88	0.18^*c*^		
(3) Own car use (α = 0.86)	3.09	1.35	0.32^*c*^	−0.10^*c*^	
(4) Own eco-mobility (α = 0.78)	2.72	0.95	−0.14^*c*^	0.44^*c*^	−0.35^*c*^
**Cycling Cities (*N* = 120)**	M	SD	1	2	3
(1) Car use-related descriptive social norm (α = 0.76)	4.34	0.74			
(2) Eco-mobility-related descriptive social norm (α = 0.88)	3.78	0.88	0.37^*c*^		
(3) Own car use (α = 0.84)	3.09	1.35	0.31^*b*^	−0.08	
(4) Own eco-mobility (α = 0.78)	2.72	0.95	−0.08	0.37^*c*^	−0.30^*c*^
**Car Cities (*N* = 121)**	M	SD	1	2	3
(1) Car use-related descriptive social norm (α = 0.80)	4.52	0.81			
(2) Eco-mobility-related descriptive social norm (α = 0.83)	3.01	0.83	−0.17^*a*^		
(3) Own car use (α = 0.84)	3.55	1.32	0.37^*c*^	−0.33^*c*^	
(4) Own eco-mobility (α = 0.79)	2.03	0.82	−0.27^*c*^	0.47^*c*^	−0.66^*c*^
**Transit metropolises (*N* = 122)**	M	SD	1	2	3
(1) Car use-related descriptive social norm (α = 0.84)	4.06	0.98			
(2) Eco-mobility-related descriptive social norm (α = 0.81)	3.74	0.75	0.38 ^*c*^		
(3) Own car use (α = 0.88)	2.46	1.38	0.26^*a*^	−0.10	
(4) Own eco-mobility (α = 0.69)	2.92	0.82	−0.09	0.26^*c*^	−0.15
**Transit cities with multimodal potential (*N* = 121)**	M	SD	1	2	3
(1) Car use-related descriptive social norm (α = 0.67)	4.39	0.77			
(2) Eco-mobility-related descriptive social norm (α = 0.87)	3.31	0.88	0.15		
(3) Own car use (α = 0.85)	3.25	1.38	0.34^*c*^	−0.18^*a*^	
(4) Own eco-mobility (α = 0.82)	2.58	0.95	−0.12	0.55^*c*^	−0.35^*c*^
**Walking cities with multimodal potential (*N* = 120)**	M	SD	1	2	3
(1) Car use-related descriptive social norm (α = 0.73)	4.33	0.77			
(2) Eco-mobility-related descriptive social norm (α = 0.83)	3.44	0.79	0.07		
(3) Own car use (α = 0.87)	3.08	1.38	0.28^*b*^	−0.22^*a*^	
(4) Own eco-mobility (α = 0.76)	2.67	0.82	−0.18^*a*^	0.50^*c*^	−0.32^*c*^
**Transit cities (*N* = 121)**	M	SD	1	2	3
(1) Car use-related descriptive social norm (α = 0.85)	4.35	0.83			
(2) Eco-mobility-related descriptive social norm (α = 0.86)	3.66	0.79	0.12		
(3) Own car use (α = 0.85)	3.11	1.42	0.33^*c*^	−0.09	
(4) Own eco-mobility (α = 0.77)	2.74	0.88	−0.10	0.30^*b*^	−0.31^*b*^

*a ¡ 0.05; b ¡ 0.01, c ¡ 0.001.*

By demonstrating that living in cities with different transport-related infrastructures is reflected in respective differences in the individually perceived mobility-related descriptive social norms, as well as the self-reported transportation means use, our results confirm the validity of Klinger’s city typology. Furthermore, in this study, the correlations between perceived mobility descriptive social norms and self-reported mobility behavior are substantive and have the expected correlation direction (Table 1): Perceived car use-related descriptive social norm correlates positively with self-reported car use (*r* = 0.317, *p* < 0.001), and descriptive eco-mobility descriptive social norm correlates negatively, albeit at a low level, with self-reported car use (*r* = −0.100, *p* < 0.001). Table 2 presents the results of a hierarchical regression analysis with self-reported car use as the dependent variable. In a first step, the two mobility-related descriptive norms were added as predictors to the regression equation. Both descriptive norms are statistically significantly associated with self-reported car use. However, the association between car use social norm and individual car use is much stronger (β = 0.35) than the association between eco-mobility social norm and self-reported car use (β = −0.16). Together, the two mobility-related descriptive norms explain 12% of variance for self-reported car use.

**TABLE 2:** (Study 1) Comparing the predictive power of “eco-norm” vs. “car-norm,” car availability and seasonal ticket for PT on self-reported car use and dummy variables of the city types (Study 1, regression analysis, *N* = 725).

Explained variable: self-reported own car use									

	Model 1			Model 2			Model 3		
			
Variable	B	SE	ß	B	SE	ß	B	SE	ß
Eco-norm	−0.26	0.06	−0.16^*c*^	−0.03	0.05	−0.02	0.01	0.05	0.00
Car norm	0.59	0.06	0.35^*c*^	0.27	0.05	0.16^*c*^	0.24	0.05	0.14^*c*^
Car availability				0.56	0.03	0.61^*c*^	0.55	0.03	0.60^*c*^
Seasonal PT ticket				0.37	0.08	0.13^*c*^	0.35	0.08	0.12^*c*^
Cycling city dummy							0.22	0.13	0.06
Car city dummy							0.39	0.13	0.10^*a*^
Transit multimodal dummy							0.31	0.13	0.08^*a*^
Walking multimodal dummy							0.27	0.13	0.07^*a*^
Transit city dummy							0.18	0.13	0.05
*R*^2^	0.12			0.51			0.52		
*F*-Change	51.40^*c*^			192.04^*c*^			86.67^*c*^		

*a < 0.05; c < 0.001, excluded variable in model 3: transit metropolis dummy.*

In order to examine the status of the two perceived descriptive norms as independent predictors of car use, we added car availability and ownership of a seasonal PT ticket as additional predictors to the regression equation in a third step. Both variables qualify as substantive car use predictors: Adding them to the regression equation increases the explained variance of self-reported individual car use from 12 to 51%. However, even after controlling for the dominant impact of car availability [(β) = 0.61, *p* < 0.001], car use-related descriptive social norm remains a significant predictor of own car use [(β) = 0.16, *p* < 0.001]. The availability of a seasonal PT ticket also qualifies as a significant predictor of individual car use [(β) = −0.13, *p* < 0.001]. However, after controlling for this variable’s impact, perceived eco-mobility-related descriptive social norm is no longer statistically significantly associated with self-reported own car use [(β) = −0.02, *p* = 0.500]. In the last step, we added the five dummy variables representing membership to one of the six Klinger city types to the regression equation as predictors. The dummy variables representing the city type car city, transit multimodal city, and walking multimodal are statistically significantly associated with self-reported car use. As a consequence, the explained self-reported car use variance increases to a statistically significant level. However, this increase is relatively low from a substantive point of view (51 to 52%). Even in this model, perceived car use-related descriptive social norm remains the second largest influencing factor (β = 0.15, *p* < 0.001), aside from car availability (β = 0.59, *p* < 0.001).

**FIGURE 3:**
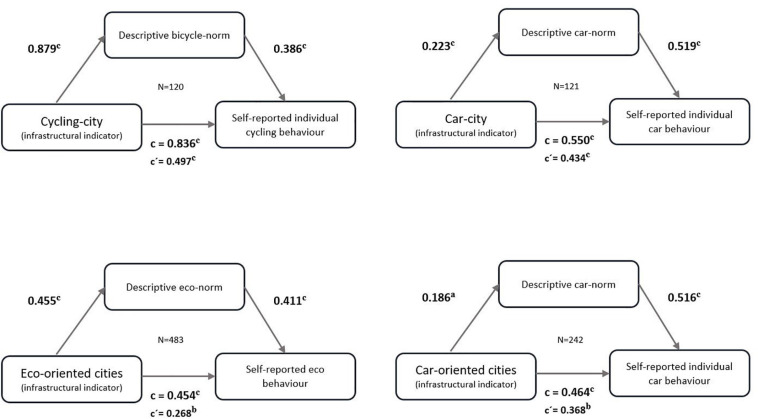
Results of the mediation analyses using PROCESS, a < 0.05; b < 0.01; c < 0.001.

The results of the last regression model reported in Table 2 already provide some empirical support that the impact of the objective transport infrastructure differences on individual mobility behavior is not a direct one, but an indirect one. For a more direct test of this assumption, we use the SPSS package PROCESS (Hayes, 2018) to conduct a respective mediation analysis. The SPSS procedure uses linear least squares regression analysis to estimate non-standardized path coefficients representing the specified indirect, direct, and total effects between three variables. The confidence intervals and inference statistics are calculated by bootstrapping with 5,000 iterations and heteroscedasticity-consistent standard errors (Davidson and MacKinnon, 1993). The aim of these PROCESS analyses is, therefore, to test to which extent descriptive norms can be seen as a mediator of the infrastructure–mobility behavior relationship. We conducted the mediation analyses (Figure 3) to investigate whether the direct relationship between the bicycle cities and bicycle use, as well as car cities and car use, remains significant after the corresponding descriptive norms are integrated into the model as a mediator. In order to gain a larger number of participants confirming these results, we have also conducted this with cycling norm as a mediator for the cities with the highest mean values of cycling behavior (*N* = 510, cycling cities, walking cities with multimodal potential, transit metropolises, and transit cities), and with car norm as a mediator and the highest mean values in car use (*N* = 240, car-oriented cities and transit cities with multimodal potential). The objective indicators of the analysis by Klinger et al. (2013) confirm this division. Therefore, this analysis aims to determine the mediating effect of the descriptive norm variables on the infrastructure–mobility behavior relationship.

In all cases, the infrastructural indicator (specific city type) predicts the self-reported mobility behavior in the way Klinger’s city typology indicates it. In other words, e.g., living in a bicycle city has a significant influence on one’s own bicycle use. This confirms the findings of the previous analyses. However, after including descriptive social norms as a mediator in the model, the infrastructural indicators predict the mediators significantly in all cases. At the same time, the descriptive norms predict, again significantly, self-reported mobility behavior patterns. However, in all PROCESS analyses, the infrastructure–mobility behavior relationship is not entirely but at least partially mediated by descriptive social norms. The connection between the infrastructural indicator and self-reported mobility behavior remains significant, albeit to a weaker extent.

#### Discussion

Using the data of a large, heterogeneous sample, study 1 finds substantive and statistically significant associations between perceived mobility-related descriptive norms and self-reported individual transport means use. Furthermore, differences in the objective transport infrastructures of cities are reflected in respective differences in participants’ perceived mobility-related descriptive social norms as well as their self-reported transportation means use. Study 1 uses the Klinger typology for identifying six city types with different mainly transport-related infrastructural characteristics. By showing that living in cities with different transport-related infrastructures is reflected in respective differences in the individually perceived mobility-related descriptive social norms as well as the self-reported transportation means use, study 1 results not only confirm the validity of Klinger’s city typology but also provide empirical evidence that the perceived mobility-related descriptive social norms mediated at least partially the effect of different infrastructural conditions on participants’ self-reported mobility behavior.

### Study 2

The central aim of study 2 is to test hypothesis H2 that social norms are embedded in environmental cues. More precisely, we assume that adding a new cycling-related infrastructural cue (here: pop-up bike lane) to a photo causes participants to perceive a significantly stronger descriptive social norm that cycling is an adequate behavior in the depicted situation than using the same photo without such a cycling-related infrastructural cue. At the same time, the experimental designs also allow us to test the alternative hypothesis H3 that differences in the perceived mobility-related descriptive social norms reflect participants’ own past transport means use rather than differences in objective infrastructural features.

#### Procedure

Inspired by Raghoebar et al. (2019), we designed an online experimental photo study by using Adobe Photoshop to add an infrastructural cue, a cycling lane similar to the temporary pop-up bike lane discussed above into a photo of a real street scene in a medium-sized German city (Figure 2). Aside from the infrastructural cue pop-up bike lane, we also added cyclists using the pop-up bike lane to the picture in Photo B. Thus, both photos in Figure 2 contain the two sources theoretically assumed to provide information about the prevailing mobility-related descriptive social norm: the infrastructural cues and fellow citizens who primarily cycle vs. use cars.

The online experiment was started with an item assessing participants’ current actual car use frequency (“How often do you personally use a car as a driver?”). Participants then read the following introduction text: “*We would like to conduct a thought experiment with you: Imagine you have to move spontaneously to a new city. You have rented the apartment online at short notice in order to find accommodation quickly. You do not know the district and the people who live there yet. Now you go out of your new apartment onto the street for the first time and the following picture awaits you. Please take your time to look at it calmly*.” Then, participants click on a button with the inscription “*Next to the view out of the window of my new flat.*” After pressing the button, Photo A or B (Figure 2) was presented. There was a timer set to ensure that the participants actually looked at the photo for at least 8 s. Next, we asked the respondents to state what they would do on their first day in their hypothetical new home (“So you live here now. What are you doing on your first day in this new district?”). Participants then completed items assessing the perceived mobility-related descriptive social norms derived for viewing Photo A or B.

#### Measurement

In study 2, we use only one item (“How frequently do you use a car?”) for assessing participants self-reported car use behavior. The items used to measure the mobility-related descriptive norm correspond to those of the first study.

#### Sample

*Via* an online access panel provider, we recruited a total sample of *N* = 110 willing to participate in the experiment. The median age of the total sample was 44.5 years (range 18–69 years); 62% were women, 51.8% used a car (almost) daily and 15.8 (almost) never; 43.9% were working full-time; 12.3% have the lowest German school-leaving certificate and 35.1% the highest possible certificate. These participants were then randomly assigned to one of two different photomontages (55 participants each).

#### Results

As a first step, we analyzed the qualitative material participants produced when confronted with the hypothetical question of what they would do on their first day in their new home. We categorized the qualitative material in two ways: (a) whether the reported behavior had an explicit reference to mobility and (b) whether the behavior was directly related to cycling or driving. This coding procedure was guided by the assumption that participants perceive and interpret the presented photos in a mobility-related way: Photo A as indicating that, in the presented street scene, car use is the “normal” most frequent behavior and Photo B as indicating that, in the presented street scene, cycling is the “normal” most frequent behavior. As expected, almost half of the respondents (49%) mentioned behaviors with a clear reference to one of the four modes of transportation means: car, public transport, walking, and cycling. In a second step, we create a variable including the information whether the mentioned mobility-related behavior is related to cycling or car use. In fact, cycling was mentioned 12 times, exclusively by people who received the photo with the pop-up bicycle lane. This represents 21.82% of all participants who looked at Photo B. Driving a car was only once mentioned by a person who looked at the photo without the pop-up bicycle lane. These results provide qualitative evidence that participants derive behavior-related expectations from observed infrastructure-related visual cues.

Turning to the quantitative test of H2, Table 3 presents the means, SDs, and correlations of self-reported car behavior and the mobility-related descriptive social norms participants derived from Photo A vs. Photo B. As already indicated by the huge mean differences of the mobility-related descriptive social norms presented in Table 3, the results of the respective *t*-tests are significant [car use-related descriptive social norm: 95% CI (−1.11, −0.36), *t*(108) = −3.902, *p* < 0.001; eco-mobility-related descriptive social norm: 95% CI (0.47, 1.11), *t*(108) = 4.866, *p* < 0.001].

**TABLE 3:** (Study 2) Labels, Cronbach’s α, means, SDs, and correlations of the descriptive norms and individual mobility behavior mean value indices (*N* = 115).

Complete Sample (*N* = 115)	M	SD	1	2
(1) Car use-related descriptive social norm (α = 0.85)	3.58	1.06		
(2) Eco-mobility-related descriptive social norm (α = 0.89)	3.58	0.93	−0.33^*c*^	
(3) Own car use (single-item measure)	3.89	1.48	0.16	−0.08
**Photo (A) without pop-up bicycle lane (*N* = 60)**	M	SD	1	2
(1) Car use-related descriptive social norm (α = 0.84)	3.95	0.83		
(2) Eco-mobility-related descriptive social norm (α = 0.89)	3.18	0.89	−0.28^*a*^	
(3) Own car use (single-item measure)	4.02	1.47	0.18	0.8
**Photo (B) with pop-up bicycle lane (*N* = 55)**	M	SD	1	2
(1) Car use-related descriptive social norm (α = 0.82)	3.21	1.13		
(2) Eco-mobility-related descriptive social norm (α = 0.86)	4.00	0.80	−0.18	
(3) Own car use (single-item measure)	3.75	1.49	0.11	−0.18

*a ¡ 0.05; c ¡ 0.001.*

The calculated effect size measure Cohen’s *d* indicates a strong effect of the intervention photo manipulation on the mobility-related descriptive social norms reported by the participants for Photo A vs. Photo B: for the car use-related descriptive social norm, Cohen’s *d* = 0.744, and for eco-mobility-related descriptive social norm, Cohen’s *d* = −1.354. Furthermore, the dummy variable representing assignment to Photo A vs. Photo B correlates significantly with eco-mobility-related descriptive social norm (*r* = 0.26, *p* < 0.001), as well as the car use-related descriptive social norm (*r* = −0.30, *p* < 0.001). For testing H3, that differences in the perceived mobility-related descriptive social norms reflect more participants’ own past transport means use than differences in objective infrastructural features, we calculated the correlation between participants’ self-reported actual car use and the reported mobility-related descriptive social norm derived from the two photos. In the present experiment, all these correlations are statistically insignificant (e.g., the correlation between actual car use and car use-related descriptive social norm is *r* = 0.16, *p* = 0.10). These results provide no empirical evidence for H3.

#### Discussion

Study 2 results provide qualitative evidence that the infrastructure-related visual cues presented by a photo activate mobility-related behavioral expectations. Furthermore, study 3 provides strong experimental evidence for H2 postulating that the perceived descriptive social norms are derived from the visual cues participants process when observing a photo: Participants who observe Photo A without the pop-up bike lane and without cyclists reported a much stronger car use-related descriptive social norm, whereas participants observing Photo B including the pop-up bike lane and cyclists reported a much stronger eco-mobility-related descriptive social norm. At the same time, the results do not support H3: In the experiment, one’s own car use behavior is not significantly associated with the mobility-related descriptive social norms participants derived for the photos.

### Study 3

Study 3 pursues two goals: (1) Replicating the test of H2, that participants derive their perceived mobility-related descriptive social norms from observed visual cues, with a second experiment. (2) Testing H4, i.e., that a photo combining infrastructural cues and observable mobility behavior of fellow citizens has as stronger influence on participants’ perceived mobility-related descriptive social norms than observing a photo presenting only infrastructural cues like the pop-up bike lane.

#### Procedure

**FIGURE 4:**
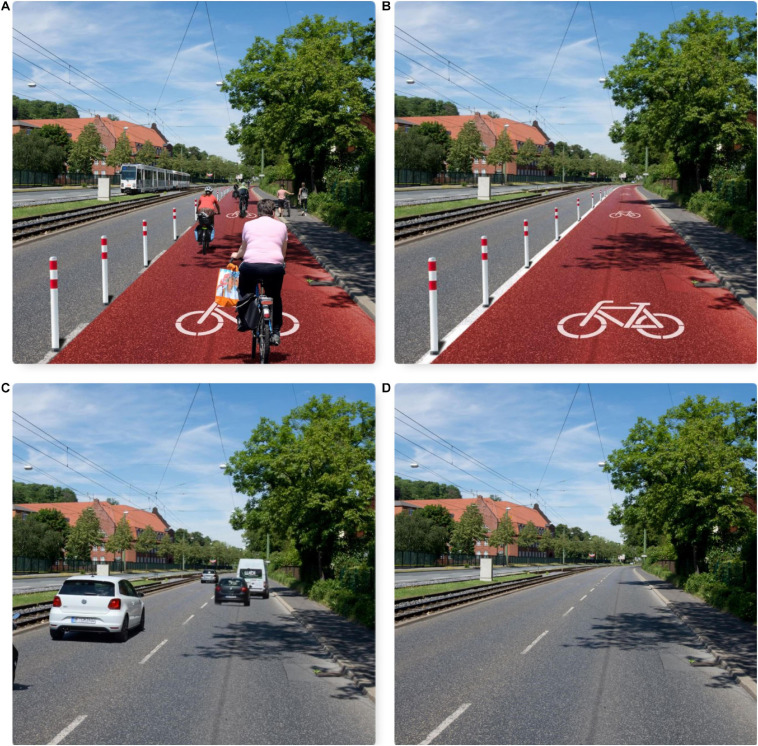
Photomontages used for experimental online study 3. On the left, infrastructure and behavior are pro-eco **(A)** and pro-car **(C)**, respectively. On the right, only infrastructure without behavior [pro-eco **(B)** or pro-car **(D)**] is shown. (Photos: Michael Motyka).

Study 3 was again carried out as an online experiment implemented by a commercial access panel provider. In contrast to study 2, four different photos are used as experimental stimuli in this experiment. The four photos were created by cross-tabulating the two factors (a) visible infrastructure (pop-up bike lane vs. no pop-up bike lane) and (b) visible mobility behavior (cyclists/car user vs. no cyclists/car user). The four photomontages resulting from this cross-tabulation are presented in Figure 4. The design and processing of the experiment were the same as in study 2.

#### Measurement

In Study 3, self-reported car use and the perceived mobility-related descriptive social norms for commuting, shopping, and everyday trips were assessed with the same items as in the two previous studies; participants again answered the hypothetical question what they would do on their first day in their new home.

#### Sample

The total sample consists of 276 individuals, who were randomly assigned to one of the four photomontages (between 65 and 71 participants each). The sample’s sociodemographic characteristics are similar to those of the sample participating in study 2: The median age is 44 years (range 18–71 years); 53% were female; 13% have the lowest and 28% the highest educational degree; 46% are working full-time. Here, 21% use the car (nearly) never and 50% (almost) every day.

#### Results

In their answers to the hypothetical question what they would do on their first day in their new home, 123 of the 276 participants (44.6%) mentioned a mobility-related behavior. Cycling was mentioned 26 times; however, again only by those presented with Photo A or B. Of the 65 participants presented with Photo A (pop-up lane and cyclists), 15 (23.08%) mentioned cycling as an activity, while of the 70 participants viewing Photo B (only pop-up lane), only 11 (15.72%) mentioned cycling.

Separately for Photos A to D, Table 4 presents the means, SDs, and correlations of self-reported car use and the mobility-related descriptive social norms participants derived from observing the four photos. A comparison of the mean values in Table 4 reveals interesting results: In the case of the car use-dominated Photos C and D, the perceived car use-related descriptive social norm is rated higher where the photo includes only infrastructural cues (Photo D). In the case of eco-mobility-dominated Photos A and B, on the other hand, the perceived eco-mobility-related descriptive social norm is rated higher when the photo presents the infrastructural cue pop-up lane plus the mobility behavior of fellow citizens (Photo A).

**TABLE 4:** (Study 3) Labels, Cronbach’s α, means, SDs and correlations of the descriptive norms and individual mobility behavior mean value indices (*N* = 276).

Complete Sample (*N* = 276)	M	SD	1	2
(1) Car use-related descriptive social norm (α = 0.85)	3.86	1.00		
(2) Eco-mobility-related descriptive social norm (α = 0.87)	3.50	0.86	−0.31^*c*^	
(3) Own car use (single-item measure)	3.70	1.61	0.18^*b*^	−0.03
**Photo (A) pop-up bicycle lane with behavior (*N* = 65)**	M	SD	1	2
(1) Car use-related descriptive social norm (α = 0.84)	3.45	1.05		
(2) Eco-mobility-related descriptive social norm (α = 0.87)	3.82	0.80	−0.24	
(3) Own car use (single-item measure)	3.55	1.67	0.22	−0.21
**Photo (B) pop-up bicycle lane without behavior (*N* = 70)**	M	SD	1	2
(1) Car use-related descriptive social norm (α = 0.84)	3.65	0.93		
(2) Eco-mobility-related descriptive social norm (α = 0.85)	3.64	0.74	−0.27^*a*^	
(3) Own car use (single-item measure)	3.94	1.45	0.17	0.04
**Photo (C) car lanes with behavior (*N* = 70)**	M	SD	1	2
(1) Car use-related descriptive social norm (α = 0.85)	4.06	0.98		
(2) Eco-mobility-related descriptive social norm (α = 0.86)	3.39	0.89	−0.20	
(3) Own car use (single-item measure)	3.61	1.70	0.36^*c*^	−0.23
**Photo (D) car lanes without behavior (*N* = 71)**	M	SD	1	2
(1) Car use-related descriptive social norm (α = 0.84)	4.23	0.84		
(2) Eco-mobility-related descriptive social norm (α = 0.86)	3.18	0.86	−0.28^*a*^	
(3) Own car use (single-item measure)	3.69	1.61	0.00	0.27^*a*^

*a ¡ 0.05; b ¡ 0.01; c ¡ 0.001.*

Using ANOVAS (Table 5), we tested the statistical significance of observed mean differences in the mobility-related descriptive norms reported for Photos A to D. The eco-mobility-related descriptive social norm [*F*(3,272) = 7.78, *p* < 0.001, η^2^ = 0.070] and the car use-related descriptive social norm [*F*(3,272) = 9.57, *p* < 0.001, η^2^ = 0.096] differ statistically significantly between the four photos. The Tukey *post hoc t*-tests indicate no significant mean differences of the two car use-dominated Photos C and D, as well as the two eco-mobility-dominated Photos A and B. Interestingly, also the *t*-test of the eco-mobility-dominated Photo B (pop-up lane and cyclists) and the car use-dominated Photo C (only infrastructure) is statistically insignificant. For all other comparisons, Tukey *post hoc t*-tests indicated statistically significant mean differences (*p* < 0.001).

**TABLE 5:** (Study 3) Tukey simultaneous tests for differences of means (ANOVA analyses, *N* = 725) between photos of Figure 4.

Dependent variable: car use-related descriptive social norm				
**Photo-Pairs**	**Difference of Means**	**SE of Difference**	**95% CI**	**Adjusted *p* Value**

C–D	−0.17	0.16	(−0.58, 0.25)	0.72
C–A	0.61^*c*^	0.16	(0.18, 1.03)	0.00
C–B	0.40	0.16	(−0.01, 0.82)	0.06
D–A	0.77^*c*^	0.16	(0.35, 1.20)	0.00
A–B	−0.20	0.16	(−0.63, 0.22)	0.61

**Dependent variable: eco-mobility-related descriptive social norm**				

**Photo-Pairs**	**Difference of Means**	**SE of Difference**	**95% CI**	**Adjusted *p* Value**

C–D	0.21	0.14	(−0.15, 0.57)	0.42
C–A	−0.43^*a*^	0.14	(−0.79, −0.06)	0.02
C–B	−0.24	0.14	(−0.60, 0.12)	0.31
D–A	−0.45^*b*^	0.14	(−1.00, −0.27)	0.01
A–B	0.18	0.14	(−0.19, 0.55)	0.58

*a ¡ 0.05; b ¡ 0.01; c ¡ 0.001.*

In study 3, we again find no statistically significant correlation between self-reported car use and the eco-mobility-related descriptive social norm. However, in study 3, there is a statistically significant, however, small (*r* = 0.18, *p* < 0.05), correlation between participants’ self-reported car use and their perceived car use-related descriptive social norm. This result provides some empirical evidence that a person’s past use of a means of transport has an impact on the car use-related descriptive social norm derived from the photos (H3).

For an additional quantitative test of H4, we created dummy variables representing participants’ assignment to one of the four photos, with Photo C as a reference category. In a regression analysis with car use-related descriptive social norm as the dependent variable and the three dummies as predictors, the dummy representing assignment to Photo D must be excluded from the regression equation due to a high multi-correlation. Both dummies representing assignment to one of the eco-mobility-related Photos A and B are significantly associated with perceived car use-related descriptive social norm [(β*_*A*_* = −0.33, *p* < 0.001, β*_*B*_* = −0.25, *p* < 0.001)].

##### Discussion

In study 3, participants’ answers to the hypothetical question what they would do on their first day in their new home replicate the statements given by study 3 participants: Again, cycling was only mentioned by those viewing a photo including the cycling-related infrastructural cue pop-up lane. This result provides further qualitative evidence for H2, i.e., that participants derive their perceived mobility-related descriptive social norms from visual cues. The result that the percentage of answers mentioning cycling further increases when participants view a photo combining the infrastructural cue pop-up lane with people cycling provides first qualitative evidence for H4. Therefore, observing a photo including the mobility behavior of fellow citizens besides infrastructural cues has a stronger influence on participants’ perceived mobility-related social norms than a photo with only an infrastructural cue. The quantitative data support this conclusion: Photo A, combining the infrastructural cue pop-up lane with the observable cycling of fellow citizens, elicits the highest perceived eco-mobility-related social norm. Obviously, infrastructural cues alone have less influence than their combination with observable user behavior. These quantitative results provide strong support for H4. Interestingly, however, for the case of the car use-related descriptive social norm, the opposite holds true: Participants assigned to Photo C (only car use infrastructural cues) reported the highest car use-related descriptive social norm. *Post hoc* discussion of this result with individuals not participating in study 3 provides a possible explanation of this result: In contrast to Photo D, Photo C was interpreted as depicting optimal car-using conditions: A “free street,” allowing for fast car driving without the need to pay careful attention to other traffic participants, neither other car users nor cyclists or pedestrians. Another result of study 3 consists of the statistically significant albeit low-level correlation between individual past car use and the car use-related descriptive social norm perceived in the photos. This finding provides some support for H3.

### General Discussion

The three studies reported on in this paper were inspired by the observation that the temporary introduction of so-called “pop-up bike lanes” implemented by municipalities with minimal investment costs for reducing cyclist threat of a COVID-19 virus infection has unintended, albeit highly welcomed from an environmental point of view, consequences: A significant increase of the number of cyclists on at least some of these “pop-up bike lanes.” This observation raises the question: How can this change be explained? The present paper postulates that the formation and activation of mobility-related descriptive social norms constitute a central mechanism, mediating between perceiving environmental cues and one’s own transport means use. However, the empirical evidence supporting this mediation mechanism raises a new question: What kind of infrastructural and/or social cues dominate the activation or formation of the social norm about which mode of transport is most adequate to use in this situation? In a first step, we conducted a correlational study aimed at providing empirical evidence for this association. The study shows substantive correlations between mobility-related descriptive norms and self-reported transport means use. Beyond that, study 1 analyzed this association in six different city types derived from a typology developed by Klinger et al. (2013). Data analysis not only confirms the mobility-related descriptive norms, self-reported transport means use correlation for all six city types, but also shows the expected significant difference in mobility-related descriptive norms as well as self-reported transport means use across the six city types. Therefore, study 1 results not only confirm the validity of Klinger’s city typology but also provide more detailed insights into the association between objective infrastructural conditions, perceived mobility-related descriptive social norms, and self-reported transport means use. The results of PROCESS mediation analyses provide some direct evidence that the association between objective infrastructural conditions and self-reported transport means use is mediated by the perceived mobility-related descriptive social norms. The two online studies focus on a more detailed analysis of the second question, i.e., what kind of infrastructural and/or social cues dominate the perception stimulating the formation of the social norm of which transport means use is most adequate in a given situation. Together, both studies provide strong evidence that, in a new situation, people use perceived infrastructural cues like the pop-up bike lane and observable user behavior like other cyclists for the formation of a mobility-related descriptive social norm, indicating that cycling is an adequate behavioral option in the presented situation. Study 3 further differentiates this insight: Combining the infrastructural cue pop-up lane with the observable cycling of fellow citizens elicited the highest perceived eco-mobility-related social norm. Obviously, in this situation, infrastructural cues alone have less influence than their combination with observable user behavior. However, it is interesting to note that the opposite is true for the descriptive social norm relating to car use. Participants assigned to a photo presenting only car use-related infrastructural cues report the highest car use-related descriptive social norm. It is possible that this photo depicting a free street indicates optimal car use conditions for most people. However, this interpretation needs further research.

#### Practical Implications

The most important practical implication of our results consists of the significance they assign to the role of mobility-related descriptive social norms as mediators of the relation between infrastructural cues and individual travel mode choice. It is likely that most transport planners and politicians are not aware how deeply they can influence peoples’ perceived descriptive social norms, i.e., their expectation of what kind of behaviors are most frequent and adequate in the designed city environment with the design of infrastructural elements such as streets or other public spaces. If policy planners and politicians are interested in promoting the inner city use of cycling and walking, our results indicate that the cooperation with designers and psychology may open a starting point for a first low-cost strategy to reach this goal: Using psychological and design knowledge for redesigning public spaces in a way that leads to eco-mobility-related descriptive social norms being formed or activated.

The second important insight one can derive from our results consists of the finding that combining a norm eliciting design with the possibility to observe people performing the behavior implied by the norm significantly increases the formation of the desired descriptive social norm. Implicitly, social movements like the Critical Mass movement are already applying this knowledge: On a regular basis, they organize events where hundreds and thousands of cyclists are “(re-)conquering” the city from cars for the purpose of cycling and walking. From a psychological point of view, with their conscious violation of traffic rules privileging car use and reserving public street space for car use, these initiatives explicitly challenge the dominant car use-related injunctive social traffic norm (Blickstein and Hanson, 2001). They are implicitly using the psychological principles explicitly researched and documented by the above described research of Keizer et al. (2011): Injunctive norm-violating behavior by others inhibits the influence of these injunctive norms. A city government interested in changing mobility-related descriptive social norms should use the Critical Mass movement as an example and officially organize events inviting the public to “(re-)conquer” newly designed and regulated public city spaces on a regular basis. The pop-up bike lanes presented at the beginning are already able to convey a certain bicycle-oriented descriptive norm. This can make them a simple, low-cost tool for city planners to change the mobility-related norms perceived by citizens and thus increase the likelihood that citizens themselves will ride their bicycles more frequently in the future. We would also like to highlight the photomontage method as a way of testing the impact of planned infrastructure measures on perceived social norms: testing which norms a planned infrastructure-related measure conveys in advance makes it possible to implement a measure adapted to the intended political goals (e.g., promoting mobility away from the car).

#### Limitations

In the present paper, the empirical evidence presented in support of H1, which states that people use the perceived infrastructural conditions and the observable travel behavior of their fellow citizens resulting from these circumstances as sources for the construction of their mobility-related descriptive social norms, is correlational in nature. Furthermore, the lab experiments provide no explicit information on how people perceive and judge the presented photos. Consequently, the question of which cues participants actually process that cause the differences in perceived mobility-related descriptive social norms remains open (H4).

Furthermore, the results reported here only focus on the role of mobility-related descriptive norms. It thus remains unclear what role mobility-related injunctive norms play in these processes, namely, whether the effects also exist through injunctive norms, or even instead of them. We would like to thank one of our reviewers for pointing out that temporarily built infrastructures like pop-up bike lanes could also be capable of triggering injunctive norms.

#### Future Research

From our point of view, one central task of future research consists of conducting more experimental intervention field studies not only providing stronger empirical evidence of the causal relation between mobility-related descriptive social norms on actual own transport means use but also systematically analyzing the role of the second norm type, i.e., injunctive social norms, on actual transport means use. Furthermore, we need a research program systematically analyzing the separate and combined impact of different infrastructure and behavior-related cues on perceived mobility-related descriptive as well as injunctive social norms. Such a research program should also include qualitative and quantitative approaches to gain a deeper insight into the kinds of infrastructural and social cues influencing the formation and activation of descriptive and injunctive social norms. The research program should also include the strategy of Keizer et al. (2011) of creating situations with contrasting mobility-related descriptive and injunctive social norms; in other words, an infrastructure that actually represents a car preference, but people who primarily use sustainable means of transport and *vice versa*. This makes it possible to differentiate more clearly what role actual norm-breaching behavior, such as parking cars on a bicycle lane, plays in influencing mobility-related descriptive social norms. Testing the influence of other materials besides photomontages is also an important future research topic regarding the usefulness of this approach for the acceptance and impact research of actual planned measures, especially materials that are more suitable for interventions in districts to promote measures, such as slogans or informative texts.

### Data Availability Statement

The raw data supporting the conclusion of this article will be made available by the authors, without undue reservation.

### Ethics Statement

Ethical review and approval was not required for the study on human participants in accordance with the local legislation and institutional requirements. The patients/participants provided their written informed consent to participate in this study.

### Author Contributions

PR contributed to the conceptualizing, methodology, investigation, formal analysis, and writing of the original draft. SB contributed to the investigation and writing of the original draft. Both authors contributed to the article and approved the submitted version.

### Conflict of Interest

The authors declare that the research was conducted in the absence of any commercial or financial relationships that could be construed as a potential conflict of interest.
